# Evaluation of a Recombinant Double Mutant of Staphylococcal Enterotoxin B (SEB-H32Q/K173E) with Enhanced Antitumor Activity Effects and Decreased Pyrexia

**DOI:** 10.1371/journal.pone.0055892

**Published:** 2013-02-06

**Authors:** Liwei Gu, Junjie Yue, Yuling Zheng, Xin Zheng, Jun Wang, Yanzi Wang, Jianchun Li, Yongqiang Jiang, Hua Jiang

**Affiliations:** 1 State Key Laboratory of Pathogen and Biosecurity, Academy of Military Medical Sciences, Beijing, China; 2 Department of Traditional Chinese Medicine Pharmacology, Shenyang Pharmaceutical University, Shenyang, China; 3 Beijing Institute of Biotechnology, Beijing, China; 4 Department of Pharmacology, Shenyang Pharmaceutical University, Shenyang, China; 5 Department of Pharmacy, Jiangsu Provincial Xuzhou Pharmaceutical Vocational College, Xuzhou, China; Aligarh Muslim University, India

## Abstract

**Background:**

Immunotherapy has been used to improve patient immune function, inhibit tumor growth and has become a highly promising method of cancer treatment. Highly agglutinative staphylococcin (HAS), a mixture of *Staphylococcus aureus* culture filtrates, which include staphylococcal enterotoxin (SE) C as the active ingredient, has been used clinically as an immunomodifier in the treatment of a number of tumors for many years. However, the use of HAS has been associated with some unavoidable side-effects such as fever. Previous studies have shown that SEB stimulates a more potent activation of T lymphocytes than SEC3, and mutations of the histidine residues eliminated the toxicity of SEB. SE mutants with decreased side-effects and/or more potent antitumor activities are required.

**Methodology/Principal Findings:**

We built a structural model of the MHC II-SEB-TCR complex and found that a mutation of SEB at Lys173 might decrease the repulsion force between the SEB-TCR, which would facilitate their interaction. From the above results, we designed SEB-H32Q/K173E (mSEB). Analysis of *in vitro* stimulation of the proliferation of human peripheral blood mononuclear cells (PBMCs), IFN-γ secretion and inhibition of the growth of various tumor cell lines demonstrated that mSEB exhibited higher antitumor activity compared with wild-type SEB (wtSEB). Notably, mSEB inhibited the growth of various tumors at an extremely low concentration with little cytotoxicity against normal cells. Three animal tumor models (C57BL/6 mouse, New Zealand rabbit and a humanized NOD/SCID mouse) were used to evaluate the *in vivo* immunotherapeutic effects. Compared with wtSEB, mSEB significantly enhanced antitumor effect in more than one animal model with reduced pyrexia toxicity and prolonged the survival of tumor-bearing mice.

**Conclusions/Significance:**

Our results suggest that SEB-H32Q/K173E retains superantigen (SAg) characteristics and enhances the host immune response to neoplastic diseases while reducing associated pyrogenic toxicity.

## Introduction

Superantigens (SAgs) are well-characterized and powerful modifiers of the immune system. As they can induce strong immune activation, SAgs have been used as biological response modifiers [1], [2]. Unlike conventional antigens and irrespective of their antigen specificities, SAgs cross-link the β chains of the variable regions of TCRs with MHC II molecules outside the peptide-binding groove without undergoing processing [3], [4]. This leads to expansion of the pool of T lymphocytes by 30% to 70% [5] and the secretion of cytokines that include IL-1, -2, -6, TNF-α and IFN-γ [6], [7], [8].

Staphylococcal enterotoxins (SEs) are well known superantigens and the most potent known activators of T lymphocytes [9]. Therefore, they have broad potential applications as immunotherapeutic agents. In China, filtrates of *Staphylococcus aureus* cultures, known as highly agglutinative staphylococcin (SEC being the active ingredient), have been used clinically as a supplementary therapeutic agent for almost 20 years [10]. However, the compliance of patients with these treatments is poor due to side-effects such as fever and local pain [11]. Therefore, the search for a feasible solution to this problem forms a significant focus of research. Recently, it has been reported that the purified SEC protein exhibits elevated SAg activity and/or reduced toxicity [12], [13], [14], [15]. In addition, previous studies have shown that SEB stimulates more potent activation of T lymphocytes than SEC3 [16].

Perabo et al. showed that SEB stimulates strong immune responses and induces tumor regression, which makes it an ideal candidate as an antitumor agent [17], [18]. Previous studies have shown that emesis is not induced by SEB with carboxymethylated histidine residues [19]. Furthermore, Korolev et al. [20] have shown that the substitution of histidine residues eliminates SEB toxicity while preserving its ability to induce T cell proliferation. These findings imply a lack of correlation between the biological activity and toxicity of SEB. Over recent decades, a striking series of advances in the knowledge of the three-dimensional structure of SAgs and of their complexes with peptide/MHC and TCRs have enabled a greater understanding of the structure-activity relationship of SEB [3], [21], [22], [23]. In order to find SEB mutants with improved tumoricidal effects and/or reduced toxicity, we focused on the structure-function relationship of SEB by constructing a model of the MHC II-SEB-TCR complex. A promising double mutant of SEB was identified and we present an initial biological activity evaluation of SEB-H32Q/K173E (mSEB).

## Results

### Molecular modeling and design

**Figure 1:**
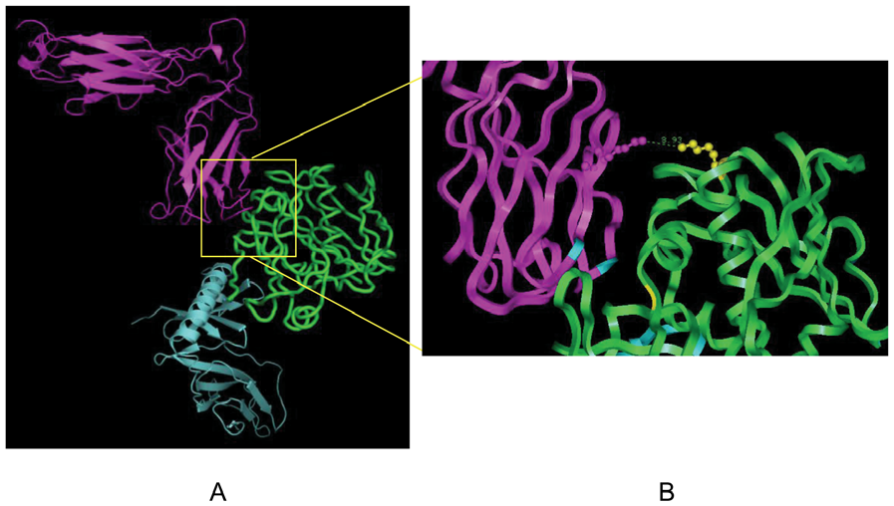
Structure of the MHC II-SEB-TCR complex. (A) The predicted model of the MHC II-SEB-TCR ternary signaling complexes. The structural complex is shown in the cartoon, SEB is colored in green, TCR is colored in magenta, and MHC II is colored in aquamarine. (B) Close views of interactions between SEB K173 and TCR K66. The two residues are shown as sticks and balls. SEB K173 is depicted in yellow and TCR K66 is depicted in aquamarine color. The distance between the two residues was 8.93 Å.

The final complex model was characterized in terms of its interactive features to improve our understanding of the mechanism of SEB recognition. Based on the predicted model of the MHC II-SEB-TCR ternary complex, we could see that SEB was situated between the MHC II and TCR molecules (Fig. 1A). From this model, Lys173 was found to be located on the area of contact where SEB binds to the TCR. The Lys173 residue of SEB was opposite to the Lys66 residue of the TCR (Fig. 1B). When the two molecules became closer, a repulsive force may form between the two positively charged residues, which would be unfavorable for SEB-TCR interactions. The substitution of Lys173 with neutral polar or negatively-charged amino acids would decrease the repulsive force between the two sites. We chose to replace the Lys173 residue of SEB with glutamic acid.

### Expression and purification of the mutant protein

**Figure 2:**
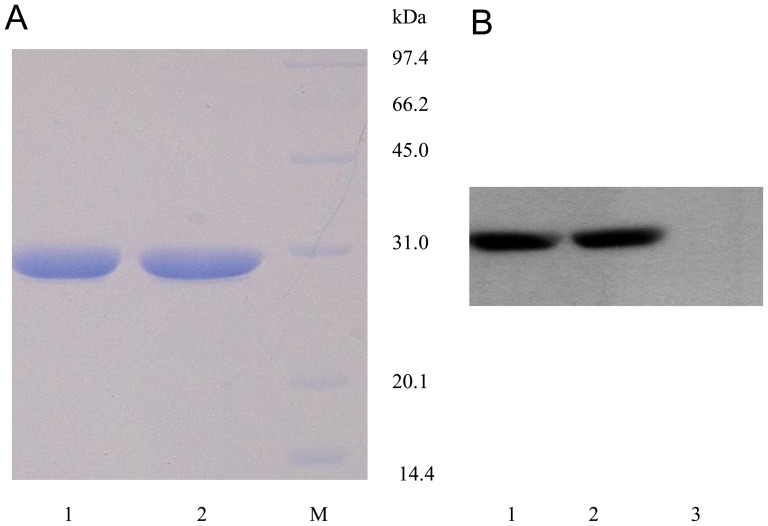
Characterization of mSEB. (A) SDS-PAGE analysis of wtSEB and mSEB. Lane 1, purified wtSEB; Lane 2, mSEB; and Lane M, low molecular weight protein marker. (B) Western blot analysis of wtSEB and mSEB using anti-SEB mAb, followed by signal enhancement using the ECL detection system. Bovine serum albumin (BSA) was used as a negative control. Lane 1, wtSEB positive control; Lane 2, mSEB; Lane 3, BSA.

Sequence analysis was conducted to verify the mSEB gene. SDS-PAGE showed that the purified protein was approximately 30 kDa, which was consistent with the molecular weight of wtSEB, and the protein purity was equal to or exceeded 95%. Expression of the mutant protein was identified by Western blot analysis using anti-SEB mouse monoclonal antibodies (Fig. 2).

### Activation of human PBMC cells

**Figure 3:**
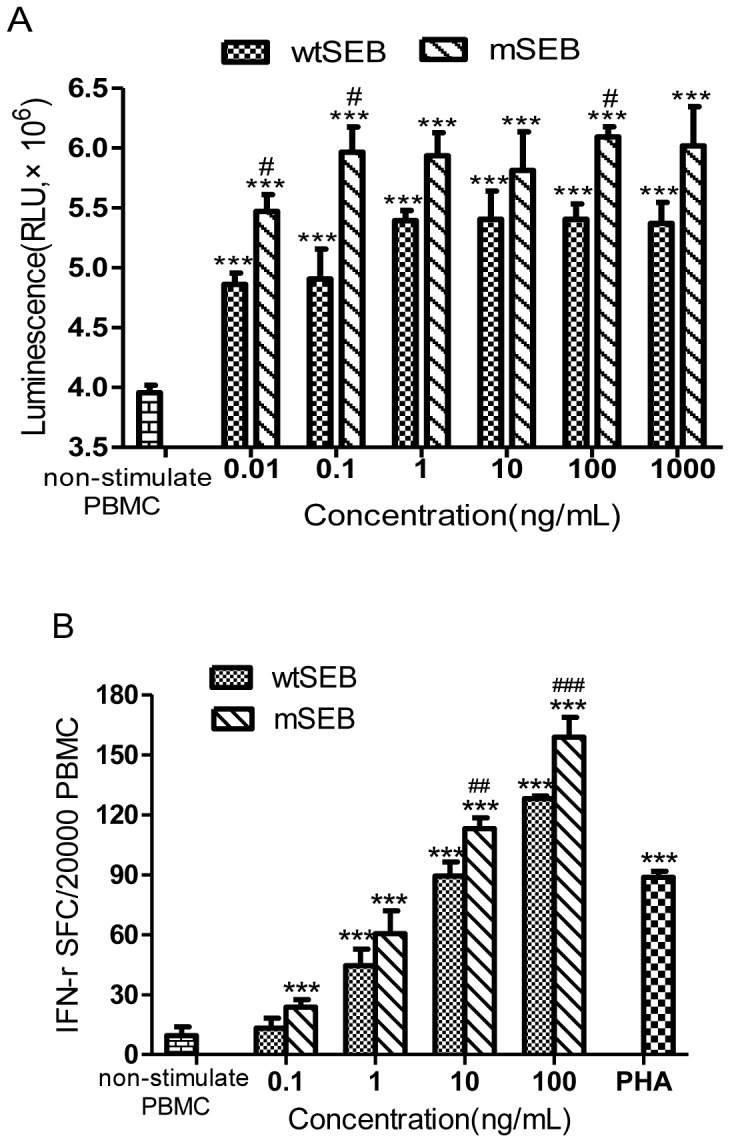
*In vitro* activation of lymphocytes. (A) Proliferation of PBMCs stimulated by wtSEB and mSEB. Comparison *versus* non-stimulated PBMCs: ^**^ *P*<0.01, ^***^ *P*<0.001. Comparison *versus* wtSEB stimulated PBMCs at the same dose: ^#^ *P*<0.05. (B) Secretion of IFN-γ by mSEB induced human T cells. Histogram of IFN-γ secreting spot forming cells (IFN-γ SFC) in PBMCs induced by different concentrations of mSEB. PHA (2.5 µg/mL) was used as positive control. Comparison *versus* non-stimulated PBMCs: ^***^ *P*<0.001. Comparison *versus* wtSEB stimulated PBMCs at the same dose: ^##^ *P*<0.01, ^###^ *P*<0.001.

The ability of SEs to stimulate the proliferation of T cells that carry specific TCR receptors is a distinctive characteristic associated with their antitumor effects. We observed that, at concentrations of 0.01, 0.1 and 100 ng/mL, mSEB stimulated the greater proliferation of human PBMCs than wtSEB (Fig. 3A).

ELISpot assays demonstrated that mSEB stimulated the secretion of IFN-γ in a dose-dependent manner (Fig. 3B). At the concentrations of 10 and 100 ng/mL, mSEB was more effective at stimulating IFN-γ secretion than wtSEB (P<0.01).

### Tumor cell growth inhibition assays in vitro

**Figure 4:**
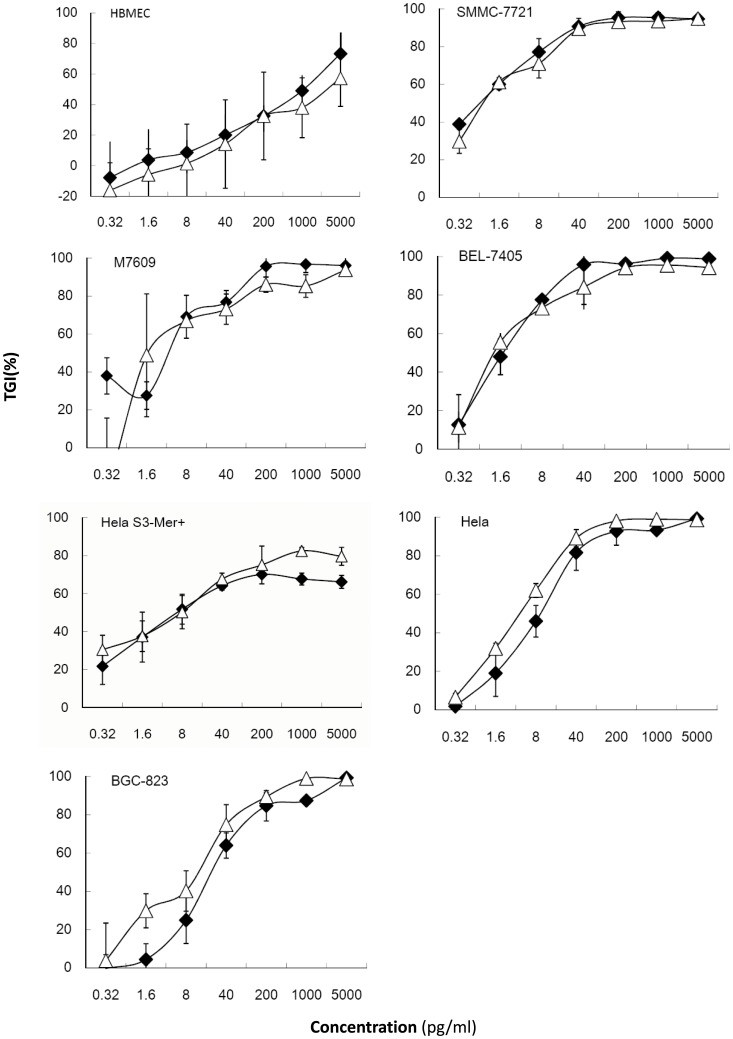
Dose response curves of the cytotoxic effects of wtSEB (-⧫-) and mSEB (-△-) in various human tumor cell lines and a normal cell line. Data represent the means of triplicate samples ± SD.

Tumor cell growth inhibition by mSEB and wtSEB was further examined *in vitro* (Fig. 4). Both mSEB and wtSEB showed wide-ranging antitumor effects, especially against hepatoma cells. In the presence of tumor cells, the IC_50_ of mSEB ranged from0.64 pg/mL to 9.7 pg/mL, while that of wtSEB ranged from 1.0 pg/mL to 33 pg/mL. Although there was no significant difference between the inhibition mediated by wtSEB and mSEB, the data from the BGC823, HeLa, and even HeLa S3-Mer^+^ cell lines indicated that the mutant exerted a slightly greater antitumor activity against these cell lines. However, in a normal cell line, mSEB only exhibited approximately 30% of the cytotoxicity exhibited by wtSEB (Table 1). Neither mSEB nor wtSEB exhibited antitumor effects in the absence of PBMCs (data not shown).

**Table 1:** The protein concentration at which the cell growth was inhibited by 50% (IC_50_).

Cell line		IC_50_ (pg/mL)	
		wtSEB	mSEB
**Normal cell line**	HBMEC	874.3	3066.4
**Hepatoma**	SMMC-7721	1.02	0.75
**Hepatoma**	BEL-7405	2.04	2.28
**Gastric carcinoma**	BGC-823	33.36	9.74
**Colon carcinoma**	M7609	5.05	4.85
**Cervical cancer**	HeLa	10.71	4.48
**Cervical cancer**	HeLa S3-Mer^+^	16.65	7.37

### In vivo tumor therapy

To investigate the potential of mSEB to activate T cells in order to boost the antitumor response, rabbits or mice were treated with various doses of mSEB after tumor inoculation. No adverse effects were observed in either species at therapeutic doses. It was observed that both mSEB and wtSEB induced a pronounced, dose-dependent antitumor effect in a C57BL/6 mice tumor model xenografted with Lewis lung tumor cells (Table 2). The antitumor effect of mSEB was better than that of wtSEB at all three comparable dosage groups. The tumor inhibition rate reached 59.64% in the 1,250 µg/kg mSEB treatment group, which was not significantly different from the wtSEB group. At doses of 250 µg/kg and 500 µg/kg, the tumoricidal effect of mSEB was much greater than that of wtSEB (*P*<0.05).

**Table 2:** The inhibition of tumor growth on Lewis lung carcinoma xenografted by mSEB.

Groups	Dose (µg/kg)	Weight of mouse (g)		Tumor weight (g)	Inhibition rate (%)
		Start	End		
PBS	—	17.96±0.79	21.14±1.00	1.593±0.534	—
	250	17.91±0.52	20.70±0.61	1.403±0.582	11.93
wtSEB	500	17.90±0.34	21.42±1.10	1.321±0.532	17.07
	1250	18.00±0.37	21.27±0.84	0.897±0.741*	43.69
	250	18.04±0.57	20.86±0.88	0.962±0.571* ^,^ #	39.61
mSEB	500	18.06±0.47	21.10±0.41	0.832±0.434*** #	47.77
	1250	17.89±0.42	20.79±0.74	0.643±0.452***	59.64

Data represent the mean ± SD (n = 10). The results shown are representative of three repeated experiments. Comparison *versus* PBS controls:

* *P*<0.05,

*** *P*<0.001.

Comparison *versus* wtSEB group at the same dose:

# *P*<0.05.

**Figure 5:**
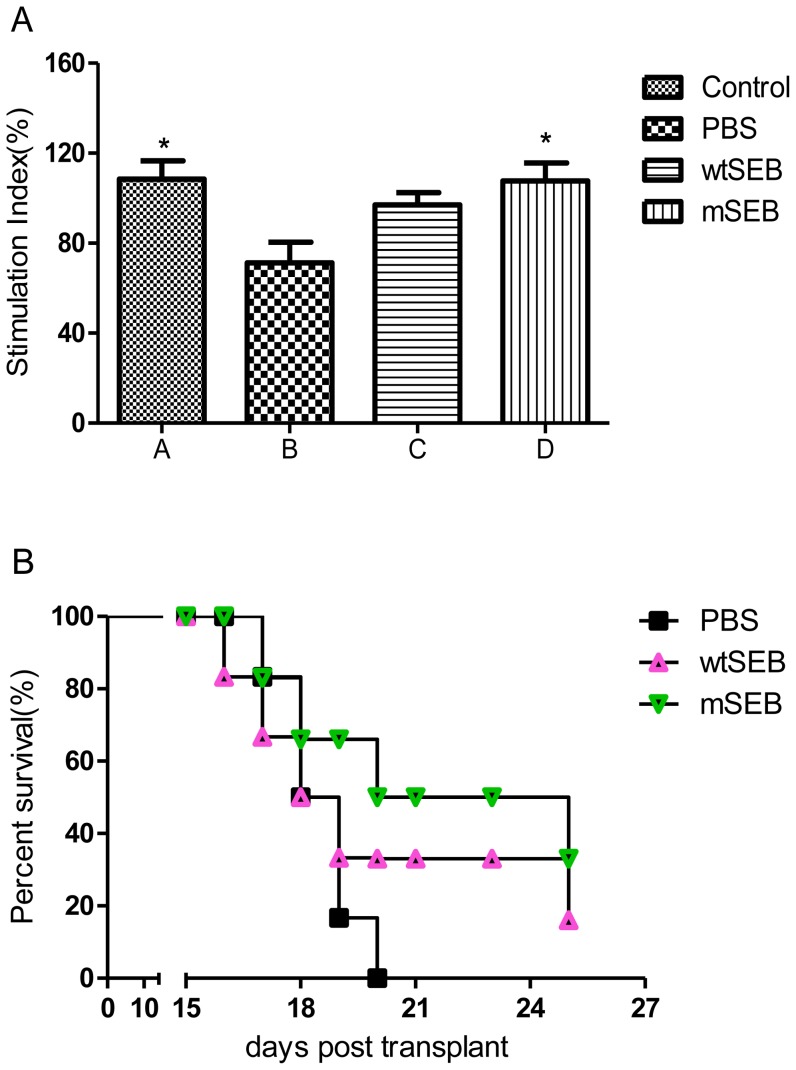
Effect of mSEB on survival rates and T lymphocyte transformation in the mouse Lewis lung carcinoma model. The tumor-bearing mice were given wtSEB or mSEB at 250 µg/kg, or PBS alone. (A) ConA-induced mouse lymphocyte transformation test (n = 6). Comparison *versus* PBS-treated group: ^*^ *P*<0.05. (B) mSEB administration protected against tumor-related mortality in mice. PBS was used as a negative control (n = 12).

The mice treated with mSEB and wtSEB had an improved immune system. However, the ConA-induced mouse lymphocyte transformation test showed a significant difference (*P*<0.05) only between the mSEB and PBS groups (Fig. 5A). In the wtSEB group, although cell proliferation was stimulated, no significant difference in induction was detected between the control and wtSEB-treated groups, while the difference between the mSEB and wtSEB groups was still not statistically significant. The survival curve of Lewis lung carcinoma-bearing mice was comparable between wtSEB and mSEB treatment groups at the overlapping dose of 250 µg/kg (Fig. 5B). The survival rate of animals in the mSEB group was higher than in the wtSEB and PBS groups, but this difference was not significant. In a humanized NOD/SCID mouse model, mSEB exerted a pronounced, dose-dependent antitumor effect. Tumor regression occurred in the three mSEB groups, but not in the wtSEB group. Notably, complete tumor regression was observed in three of the five mice treated with 450 ng mSEB (Table 3). Furthermore, at equivalent doses (150 ng), the tumoricidal effect of mSEB was significantly higher than that of wtSEB (*P*<0.05). At the end of the trial, the average body weight of PBS-treated mice had increased by 9.95%, compared with 17.84% in the 450 ng mSEB-treated mice.

**Table 3:** Inhibition of tumor growth by mSEB in xenografted NOD/SCID mice.

Groups	Dose (ng)	No. of mice		Weight of mouse (g)		Tumor weight (g)	Mean no. of tumors	Incidence	Inhibition rate (%)	
		Start	End	Start	End				i	ii
PBS	—	6	6	23.26±1.73	25.83±1.41	1.281±0.388	20.8	6/6	—	NA
PBMC	—	6	6	23.92±1.44	26.38±2.67	0.871±0.458	10	6/6	31.99	—
wtSEB	150	5	5	23.81±0.89	27.05±0.97	0.521±0.181	2.2	5/5	59.3	40.15
	50	5	5	23.90±1.58	28.28±1.55	0.542±0.360	3.4	4/5	57.69	37.78
mSEB	150	5	5	23.80±1.57	28.00±2.34	0.235±0.199** ^, ^ Δ ^, ^ #	1	4/5	84.47	77.17
	450	5	5	24.32±1.80	29.6±1.42	0.166±0.232** ^, ^ Δ	0.8	2/5	87.03	80.92

Data represent the mean ± SD. The results shown are representative of duplicate experiments. NA stands for not available. Comparison *versus* PBS controls:

** *P*<0.01.

Comparison *versus* PBMC group:

Δ *P*<0.05.

Comparison *versus* PBMC group:

# *P*<0.05.

i: the inhibition rate compare to PBS group; ii: the inhibition rate compare to PBMCs group.

**Figure 6:**
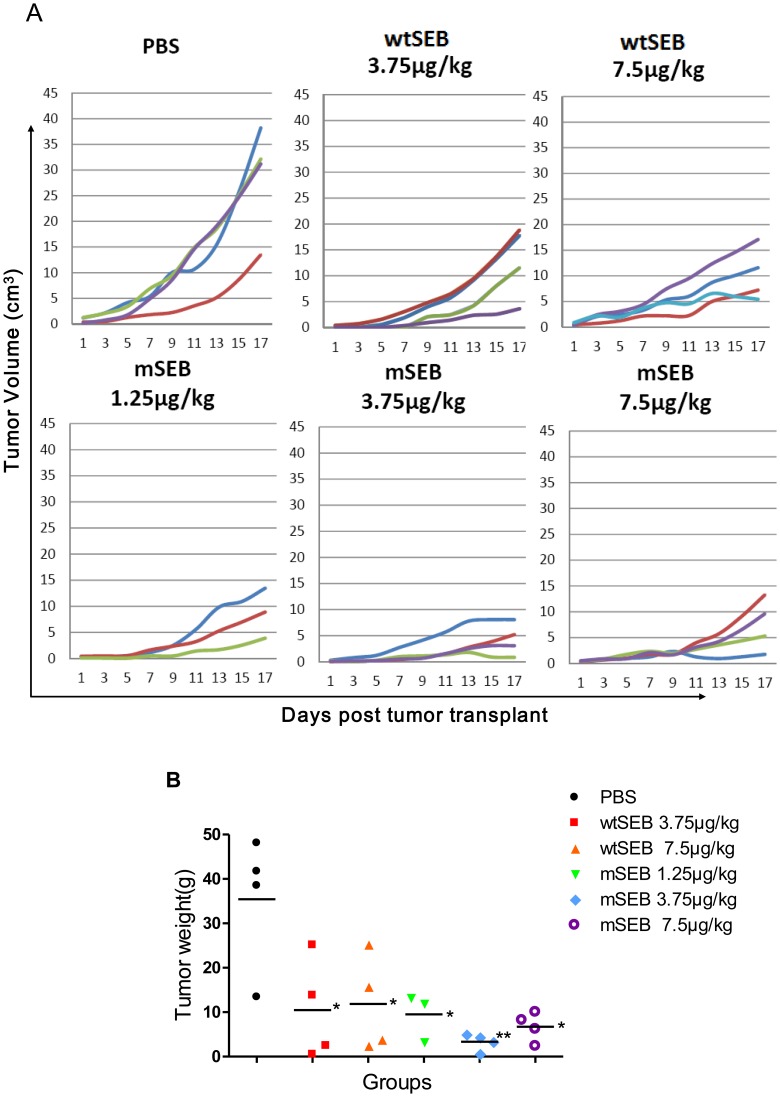
Administration of mSEB to rabbits inhibited growth of VX2 tumors. (A) Comparison of tumor growth curves of rabbits by group. Each line represents tumor growth in a single rabbit. (B) Comparison of tumor weights by group. Each symbol represents the tumor weight from a single rabbit. Horizontal lines indicate the mean tumor weight in each group. Comparison *versus* PBS controls:^*^ *P*<0.05, ^**^ *P*<0.01.

In the rabbit model, tumor growth increased exponentially in PBS-treated rabbits, whereas tumors in the treatment group grew only slightly or entered remission. Three cases of tumor remission were observed in the 3.75 µg/kg mSEB treatment group, two in the 7.5 µg/kg mSEB group and one in the 7.5 µg/kg wtSEB group (Fig. 6A). In the three mSEB treatment groups, the inhibition rate was the highest in the 3.75 µg/kg mSEB treatment group (Fig. 6B). The mutant SEB was associated with a higher inhibition rate than the wtSEB in both overlapping dosage groups (3.75 µg/kg and 7.5 µg/kg). Although similar results occurred in repeated tests, the difference was still not statistically significant.

### Toxicity as assessed by pyrexia

**Figure 7:**
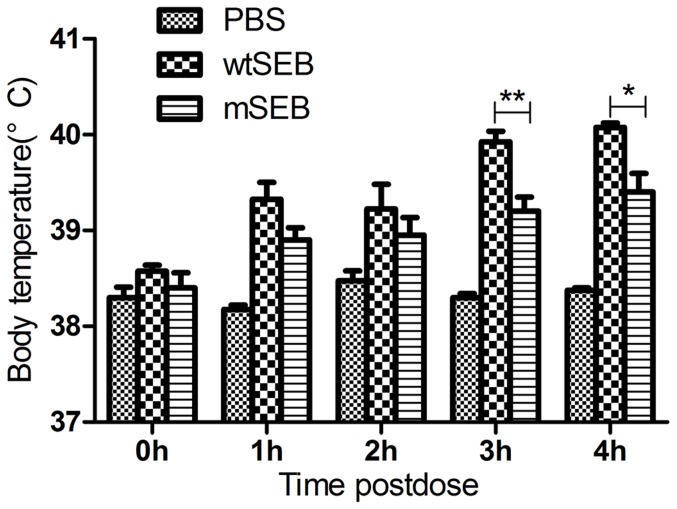
Pyrogenicity of wtSEB and mSEB proteins in a rabbit model. Rectal temperatures of rabbits (n = 4) were monitored for 4 h and the mean temperature increase calculated. PBS was used as a negative control. mSEB *versus* wtSEB group: ^*^ *P*<0.05, ^**^ *P*<0.01.

Pyrogenicity was evaluated in rabbits to investigate the influence on toxicity of the mutations introduced into wtSEB at amino acid sites 32 and 173. Fever has been defined as the body temperature increasing by more than 0.5°C over a 4 h period. In the two treatment groups, rabbits invariably developed a fever that peaked at 4 h post-dose, whereas temperatures were stable in the PBS group. During the 4 h observation period, the rectal temperature changes (body temperature) induced by mSEB were less than those induced by wtSEB. At 3 to 4 h post-dose, significant differences in body temperature were detected between the mSEB- and wtSEB-treated groups (Fig. 7).

### Clinical observations

The symptoms of mSEB poisoning in rabbits were very similar to those induced by wtSEB. No unscheduled deaths occurred in any of the groups in this study. All rabbits in the mSEB group appeared to have reduced spontaneous activity, anorexia, ptosis, and were unresponsive to outside stimuli 4 to 6 hours post-dose; one quarter of the animals developed diarrhea after 24 hours. These reactions disappeared after 48 hours. In the wtSEB group, the animals became prone, with reduced spontaneous activity, anorexia and ptosis, as well as being unresponsive to outside stimuli within 2 to 4 hours after drug administration; three quarters of the animals developed diarrhea approximately 16 hours post-dose, and these reactions gradually resolved 48 to 54 hours post-dose.

## Discussion

Tumor immunotherapy, which is based on immuno-activation, has become the fourth most promising form of antitumor therapy after surgical techniques, cancer radiotherapy and chemotherapy. SAgs are well-characterized and powerful modifiers of the immune system. Bacterial SAgs represent a unique class of microbial toxin that has evolved to target two crucial immune cell receptors, the TCR and MHC class II molecules. Although SEs induce abundant T cell activation, the development of a purified alternative with enhanced antitumor effects and reduced toxicity is required due to the side-effects associated with the clinical use of SEs. However, several encouraging reports have indicated that there is no connection between adverse effects and the efficacy of T cell stimulation [19], [24]. Just as Korolev et al. [20] proposed, we also confirmed that His 32 is an important residue that is closely related to SEB toxicity [25]. Therefore, we built the first MHC II-SEB-TCR ternary complex structural model to analyze the interaction between SEB and these receptors. This MHC II-SEB-TCR complex structure can be considered as an approximation of how SEB might activate T cells. The predicted model of the MHC II-SEB-TCR complex provides us with features that are consistent with the experimental data.

*In vitro* analysis suggested that, at very low concentrations, mSEB activated T cells and inhibited growth of a variety of human tumor cells, including hepatic, colonic, cervical and gastric cancer cells. mSEB even inhibited the growth of alkylating agent-resistant tumor cells, HeLa S3-Mer^+^, and the HeLa cell line to similar extents, which suggests that the antitumor effects of mSEB are not limited by the tumor type. IFN-γ, which is an important Th1 cytokine in tumor immunotherapy, has been used clinically for several years [26], [27]. Moreover, IFN-γ has been documented to induce MHC II expression on the tumor cell surface [28], resulting in enhanced recognition by the immune system. Encouragingly, mSEB elicited obviously more IFN-γ secretion than wtSEB at all concentrations tested, although without statistical significance, which could be highly beneficial in cancer patients. *In vitro* results demonstrated that mSEB retained the characteristics of superantigens and their immuno-active abilities.

In this study, C57BL/6 Lewis lung mice model was used to analyze the cancer immunotherapeutic potential of mSEB. It was observed that mSEB significantly inhibited the growth of Lewis lung carcinoma and furthermore, the antitumor activity of mSEB was significantly greater than that of wtSEB. Because administration of mSEB resulted in greatly reduced tumor weights at lower doses, we continue to compare the life extension and the T lymphocyte transformation at 250 µg/kg. At this dose, mSEB prolonged allograft survival in mice. The result of lymphocyte transformation analysis suggested low dose mSEB resulted in a significant increase in the frequency of transforming cells. Thus, it can be concluded that mSEB enables the host to generate antitumor immune responses by stimulating and enhancing functional immunological responses.

Normal mice reportedly displayed weak immune responses to SEB, probably because this enterotoxin does not bind strongly to murine MHC II molecules [29], [30], [31]. Although non-human primates are appropriate animal models for studying SAgs [32], [33], in the face of ethical concerns, exorbitant costs and lack of availability of suitable laboratory reagents, researchers have established a humanized severe combined immune-deficiency mouse model [34], [35]. We developed a humanized NOD/SCID mice model to analyze the antitumor effects of mSEB. Using this model we observed that, at equivalent doses, the antitumor activity of mSEB was significantly greater than that of wtSEB. Compared with administration of wtSEB, mSEB markedly reduced tumor weights, numbers and incidence rates. Furthermore, tumor regression was clearly detected in mSEB treatment groups.

It is known that rabbits are SE toxins sensitive, and the resulting cardiovascular and immune effects are similar to humans [36]. Therefore, rabbits are preferable to normal mice for evaluation of the tumoricidal effects of SE. Surprisingly, in VX2 rabbit tumor model, the dose of 3.75 µg/kg mSEB exerted the strongest antitumor effects. We then analyzed whether the antibodies against SEB were the main inhibitors of the effect of other doses. Titers of SEB antibodies were assessed in all rabbits before they commenced the experiment. SEB-specific antibodies were detected in rabbit serum 10 days after the first administration (data not shown). By the end of the experiment, we observed that lower levels of antibodies were detected in the mSEB groups, but the level of antibody produced was not dose-dependent. Rabbits in the 1.25 µg/kg and 3.75 µg/kg groups had similar levels of antibody. The molecular mechanism underlying these findings warrants further investigation. The observation that such low doses of mSEB achieved such an exceptionally good inhibition rate is encouraging in terms of broadening the therapeutic window.

Fever induction is a known clinical side-effect of SE treatment. Thus, it is necessary to investigate whether the enhanced superantigen activity identified for mSEB was also associated with increased pyrexia toxicity. The rectal temperatures of rabbits used to assess pyrexia were continuously monitored for a week prior to the formal experiment (normal range, 37.7∼39.2°C). After administration, the body temperature of rabbits in the wtSEB group increased significantly compared to the mSEB group. Furthermore, the temperature of all rabbits in the wtSEB group exceeded 40°C at 4 hours post-dose, while this did not occur in any rabbit in the mSEB group. Our assessment of fever induction in the rabbit model suggested that mSEB was less toxic in this respect than wtSEB. Although mSEB and wtSEB induced similar toxic clinical signs at the achieved maximum dosage (500 µg/kg), wtSEB appeared to cause more toxic side effects than mSEB.

The evaluation of each biological activity was equilibrated and stable, as shown by the retained superantigen characteristics and an enhanced host immune response. We believe that this evaluation is adequate for acquiring reliable and accurate information regarding the properties of this mutant. In addition to the above results, there were some limitations to this study. For example, although mSEB induced an enhanced antitumor effect, the efficacy of certain T cell subpopulations remains unclear.

In summary, this relatively complete story demonstrates that SEB-H32Q/K173E retained characteristic SAg tumoricidal activity, while the toxic side-effect of pyrogenicity was reduced. This preliminary biological activity study indicates that systemic administration of mSEB can enhance the host immune response to neoplastic diseases with acceptable toxicities and, furthermore, represents a basis for the design of more effective and less toxic cancer immunotherapy agents.

## Materials and Methods

### Ethics statement

Human blood samples were obtained from the Affiliated Hospital of Academy of Military Medical Sciences (Beijing, China). Written informed consent was received from all participants involved in the study and samples were collected with the approval of the Academy of Military Medical Sciences (AMMS) and the Affiliated Hospital. Animal experiments were conducted in accordance with the recommendations of the Guide for the Care and Use of Laboratory Animals of the National Institutes of Health and approved by the Animal Ethics Committee of the AMMS. All surgery was performed under anesthesia and all efforts were made to minimize animal suffering.

### Cell lines

The following human cell lines were obtained from the Institute of Basic Medical Sciences of Chinese Academy of Medical Sciences (Beijing, China): hepatoma SMMC-7721, BEL-7405, gastric carcinoma BGC-823, cervical cancer HeLa and a normal cell line, human brain microvascular endothelial cells (HBMEC). Human colon tumor M7609 was obtained from the Japanese Cancer Research Resources Bank (Tokyo, Japan) and the alkylating agent-resistant tumor cell line, HeLa S3-Mer+, from the American Type Culture Collection (ATCC).

All tumor cell lines were maintained in RPMI 1640 culture medium (Gibco, Gaithersburg, MD, USA), which was supplemented with 10% newborn calf serum (Biochrom, Berlin, Germany) and 1% nonessential amino acids, 2 mM L-glutamine, 100 units/ml penicillin G, 100 units/ml streptomycin, 0.01 M HEPES, and 1 mM NaHCO3. The HBMEC line was maintained in RPMI 1640 with 10% fetal bovine serum (FBS) (Gibco) and 10% Nu-Serum (BD Biosciences, Franklin Lakes, NJ, USA). All cells were incubated at 37°C in 5% CO_2_ in a humidified atmosphere.

### Animals

C57BL/6 female mice (aged 4–6 weeks) and New Zealand rabbits (female, 2.5–3.0 kg) were purchased from the Academy of Military Medical Science (Beijing, China). Severe combined immunodeficiency mice (NOD/SCID male mice, 5–6 weeks old) were purchased from Beijing HFK Bio-Technology (Beijing, China).

### Bioinformatics analysis

SAgs were determined to simultaneously bind to both the MHC II and TCR molecules on antigen presenting cells and T lymphocytes, respectively [37]. To investigate the relationship between the affinity of SEB for TCR and MHC II and its ability to activate T cells, a model of MHC II-SEB-TCR ternary signaling complex was required. Although a structural model of the MHC II-SEB-TCR ternary complex has not been reported previously, the TCR Vβ-SEB and the SEB-peptide/HLA-DR1 structural models provide an opportunity to propose a structural model for the MHC II-SEB-TCR complex. A number of studies have constructed models of MHC-SAg-TCR ternary signaling complexes for TSST-1, SEB and SpeC by the superposition of the common elements in both the SAg–MHC and SAg–TCR X-ray crystal structures [22], [37], [38], [39], [40]. We built the MHC II-SEB-TCR complex structural model according to this approach. Molecular modeling was performed in Discovery Studio 2.5 (Accelrys Inc.)

### Purification and identification

The SEB gene was engineered into pET-32a (+) (Novagen, Madison, WI, USA) and expressed in the *Escherichia coli* BL21 (DE3) strain. The protein was purified by carboxymethyl (CM)-cellulose column chromatography and verified by Western blotting. The LPS content was analyzed by the Tachypleus Amebocyte Lysate (TAL) method. Samples that contained less than 0.5 EU/µg LPS were deemed to be suitable for use in the following assays.

### Proliferation and activation of human peripheral blood mononuclear cells

Briefly, PBMCs were separated from heparinized blood obtained from healthy donors by Ficoll-Paque PLUS (GE Healthcare, Fairfield, Connecticut, USA) density gradient centrifugation, as previously described [41]. Freshly isolated PBMCs were suspended in RPMI 1640 culture medium and then distributed (10^5^ cells/well) into 96-well plates containing test samples at different concentrations. After 72 h, cell viability was detected with the Luminescent ATP Cell Viability Assay Reagent (Promega, Madison, WI, USA). Human-IFN-γ production was determined using ELISpot kits according to the instructions provided by the manufacturer (Dakewe Biotech, Beijing, China). The frequency of antigen-specific IFN-γ-secreting spot forming cells (SFC) was determined with a computer-assisted video-imaging ELISPOT reader (CTL, Cleveland, Ohio, USA).

### In vitro antitumor activity assay

*In vitro* antitumor effects were determined using an MTS assay, as previously described [42]. Five-fold serial dilutions of mSEB and wtSEB were prepared over the range of final concentrations from 5,000 to 0.32 pg/mL. Absorbance values at 490 nm were recorded using a SpectraMax Plus spectrophotometer (Molecular Devices, Sunnyvale, USA). Data are presented as the percent inhibition of tumor growth (TGI) ± SD, which was calculated as:
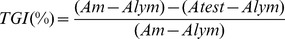
where Am represents the absorbance value of tumor cells mixed with effector cells, Alym represents the absorbance value of wells containing effector cells only and Atest represents the absorbance value of tumor cells grown in the presence of effector cells and superantigens. Each TGI value represents the average of at least triplicate samples.

### In vivo tumor therapy in normal mice

The mouse tumor model was established according a previously described protocol [43]. Lewis lung carcinoma cells (2×10^6^) were implanted subcutaneously (s.c.) into the right axilla of C57BL/6 mice on day 0. The implanted mice were randomly divided into seven groups (10 mice/group) and injected intraperitoneally (i.p.) with mSEB or wtSEB in PBS or vehicle alone on days 1, 4 and 7. Mice were weighed and examined frequently for clinical signs of adverse effects associated with treatment. Tumors were dissected out and weighed on day 11.

### T lymphocyte transformation test

C57BL/6 mice were randomly divided into five groups (n = 6) and treated with PBS, wtSEB and mSEB at 250 µg/kg. Animals were then sacrificed and spleens were removed under sterile conditions for T cell proliferation assays as previously described [44]. Cells were suspended in culture medium and the concentration adjusted to 1×10^6^ cells/mL. Samples (100 µL) were incubated in 96-well plates with 100 µL ConA (final concentration 5 µg/mL) for 48 h and absorbance values were measured by the MTS assay. The stimulation index (SI) was calculated according to the formula:
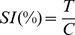
Where T represents the mean value of experimental wells, C represents the mean value of control wells.

### In vivo tumor therapy in NOD/SCID mice

A severe combined immune-deficiency mouse model described in a previous study [35] was modified slightly to assess tumor therapy *in vivo*. In brief, each mouse was injected i.p. with 5×10^6^ human liver cancer SMMC-7721 cells in 0.2 mL PBS on day 0. Each mouse (with the exception of those in the PBS group) was injected i.p. with 1.5×10^7^ PBMCs on day 2. Thereafter, all animals were injected i.p. with mSEB or wtSEB in 0.2 mL PBS or vehicle alone on three occasions at 3-day intervals. Mice were weighed and examined frequently for clinical evidence of adverse effects. On day 35, the animals were sacrificed and tumor nodules removed, counted and weighed. Each treatment cohort consisted of five to six animals.

### In vivo tumor therapy in rabbits

Squamous cell carcinoma VX2 tumor cells developed as a result of a malignant change in the cells of a Shope virus-induced skin papilloma of a rabbit. This tumor, which is highly malignant and metastasizing, was used as an additional *in vivo* tumor therapy model. Tumor tissue (approximately 0.05 g) was implanted intramuscularly (i.m.) into the right thigh of the recipient. Animals were randomly divided into five groups when the tumor diameter reached 1 cm to 1.2 cm. The left thigh of animals in the three test groups was injected i.m. with mSEB at three dose levels (1.25 µg/kg, 3.75 µg/kg and 7.5 µg/kg). The remaining three groups were used as controls, one of which received PBS and the other two received wtSEB (3.75 µg/kg and 7.5 µg/kg). Rabbits were treated three times at intervals of 3 days.

### In vivo toxicity

For the pyrexia model, only rabbits with stable body temperatures in the normal range (fluctuations of <0.5°C during a 4 h experimental period) were used to assess pyrexia induced by injections of the test proteins. The test injections were administered only after the rectal temperature of each animal had been stable for at least 1 h. Each animal was intravenously (i.v.) administered 5 µg/kg wtSEB or mSEB in PBS, or PBS alone as a vehicle control [45], and body temperature was monitored over 4 h.

Each healthy New Zealand rabbit was administered at 500 µg/kg wtSEB or mSEB intravenously, and clinical signs and toxicities were subsequently monitored.

### Statistical analysis

One-way ANOVA followed by Dunnett's tests were performed using SPSS software, version 11.0 and MS Excel. The results are presented as the mean ± SD. All tests were two-sided and the significance level was set at 0.05.
